# A72 LOCAL RECURRENCE RATES AFTER RESECTION OF LARGE COLORECTAL SERRATED LESIONS WITH OR WITHOUT MARGIN ABLATION

**DOI:** 10.1093/jcag/gwac036.072

**Published:** 2023-03-07

**Authors:** L Amar, R Djinbachian, D von Renteln

**Affiliations:** 1 Montreal University Hospital Research Center; 2 Gastroenterology, Montreal University Hospital Center (CHUM), Montreal, Canada

## Abstract

**Background:**

Endoscopic mucosal resection (EMR) has become the mainstay of therapy with regard to larger polyps. Serrated lesions (SLs) including traditional serrated adenomas (TSA), large hyperplastic polyps (HP) and sessile serrated lesions (SSLs) have been associated with high rates of incomplete resection. There has been an increasing role of margin ablation combined to EMR to reduce local recurrence.

**Purpose:**

Our aim was to evaluate local recurrence rates (LRR) after resection of large colorectal SLs and the use of margin ablation in preventing recurrence

**Method:**

Adult patients who underwent resection colorectal SSL, TSA, or HP polyps ≥ 15 mm from 2010-2019 with a colonoscopy follow-up within 18 months were identified through pathology database and electronic medical records search. Hereditary CRC syndromes, follow-ups longer than 18 months or no follow-up, surgical resection were excluded. The primary outcome was LRRs (either histologic or visual) during the first 18-month follow-up. Secondary outcomes were LRRs for polyps 15-19 mm and ≥20 mm, LRR after margin ablation for polyps ≥20mm.

**Result(s):**

Polyp resection was performed on 125 polyps in 112 patients (54.1 %women; mean age, 62.9 years). The mean size of polyps was 22.9 mm, with 58.4% ≥20mm. 76.0% of polyps were resected via EMR and 33.3% of polyps ≥20 mm received margin ablation. First surveillance colonoscopy was on average 8.4 months later and the resection scar was seen in 54.4% of lesions. Overall LRR for the first 18-months was 16% [95% confidence interval (CI) 10.5-23.6] (13% for polyps 15-19mm, 18% for ≥20mm, 22% for ≥30 mm). LRR for ≥20mm SLs was significantly lower after margin ablation (5.0% vs 24.0%; p=0.048).

**Conclusion(s):**

The local recurrence rate for SLs ≥15 mm is high with 16% overall recurrence, and increases with polyp size. Thermal ablation of the margins is superior to no ablation in reducing LRRs for ≥ 20mm SSLs.

**Please acknowledge all funding agencies by checking the applicable boxes below:**

None

**Disclosure of Interest:**

None Declared

